# Editorial: Endocrinology of loneliness and social isolation

**DOI:** 10.3389/fnbeh.2022.978386

**Published:** 2022-08-10

**Authors:** César Venero, Angela J. Grippo, Julian C. L. Lai

**Affiliations:** ^1^Department of Psychobiology, Universidad Nacional de Educación a Distancia (UNED), Madrid, Spain; ^2^Department of Psychology, Northern Illinois University, DeKalb, IL, United States; ^3^Psychophysiology Laboratory, Department of Social and Behavioural Sciences, City University of Hong Kong, Kowloon, Hong Kong SAR, China

**Keywords:** loneliness, social isolation (SI), cortisol, inflammation, immunity

Recent research has identified loneliness or perceived social isolation (PSI) as one of the most crucial factors contributing to ill health and mortality (Rico-Uribe et al., 2018; Wang et al., 2018). Loneliness is conceptually different from social isolation, which is an objective measure of the lack of social relationships and interactions (Cacioppo et al., 2014). Loneliness relates to the quality rather than the quantity of social relationships, and thus individuals can be socially content when alone or be lonely in a crowd. Due to the rising prevalence of loneliness, researchers have called for immediate public health attention to address this “behavioral epidemic” (Jeste et al., 2020). The urgency of this issue has triggered a proliferation of studies aimed at addressing the physiological mechanisms translating loneliness into ill health or increased mortality in the last decades (reviewed by Cacioppo et al., 2015; Brown et al., 2018; Campagne, 2019; Smith et al., 2020). It has been suggested that dysregulation in the functioning of the neuroendocrine-immune axis is a central feature of the pathophysiology of loneliness (e.g., Hawkley and Cacioppo, 2003), despite ambiguous findings concerning the effect of social isolation on HPA and immune functioning (Hawkley et al., 2012). According to Lai et al. (2019), glucocorticoid resistance and excessive systemic inflammation associated with this pathophysiological condition may manifest in the form of enhanced non-specific immunity and suppressed humoral immunity, which explains the increased susceptibility to inflammation-driven and infectious diseases in lonely individuals. The influence between loneliness and excessive inflammation seems to be bidirectional as experimental administration of an inflammatory challenge using endotoxin has been shown to induce a feeling of social disconnection in humans (Eisenberger et al., 2010).

This collection consists of seven papers with five empirical or preclinical and clinical studies, one review focusing on the behavioral and neurophysiological consequences of social isolation and loss (Vitale and Smith), and one perspective paper on the role of arginine vasopressin in human behaviors (Crespi et al.). In addition to reiterating findings on the association of social isolation and loneliness with altered endocrine-immune functioning or ill health, this collection may further our understanding of the complexity of this association by illuminating the effects of specific modulating factors on the health consequences of loneliness and social isolation in humans and animals. In a cross-sectional study with a mixed-sex sample of 79 seniors (mean age = 64.48 yrs., 40 women), Crespo-Sanmiguel et al. reported that loneliness was significantly associated with psychological and physical health in males, but not in females. Interestingly, these two variables had no relationship with cortisol indices including the cortisol awakening response (CAR), diurnal cortisol slope (DCS), and bedtime cortisol levels. As a result, cortisol indices did not mediate the connection between loneliness and poor health. In a separate study of 222 older adults from racially and socioeconomically diverse backgrounds (mean age = 76.82 yrs., 38% Black, 13% Hispanic), Van Bogart et al. examined the associations of loneliness—measured as a trait using a three-item loneliness scale and as an aggregated momentary feeling using an ecological momentary assessment (EMA) protocol—with basal plasmatic levels of inflammatory markers including C-reactive protein (CRP) and several cytokines, as well as LPS-stimulated levels of cytokines. A significant association was observed between trait and aggregated momentary loneliness and basal levels of CRP, a marker of systemic inflammation. This finding is consistent with the results reported in a recent meta-analytic review (e.g., Smith et al., 2020). The excessive inflammation associated with the lonely phenotype has also been verified by Nersesian et al. (2018) in a large prospective study.

Two papers in this collection focused on the behavioral and endocrine-immune effects of early-life social isolation in rodents (prairie voles) with (Sailer et al.) or without (Donovan et al.) concomitant exposure to a chronic stressor. The latter study demonstrated that social isolation had no effect on anxiety- and depression-like behaviors, but decreased the peripheral level of the pro-inflammatory cytokine IL-1β in isolated males compared to cohoused females. Social isolation also altered the density of microglia in specific regions of the brain, demonstrating that social isolation affected both peripheral and central markers of immunity. Although a main effect of social isolation on levels of IL-1β was not observed, the finding of a significant sex by treatment effect warrants further studies to get a better understanding of the impact of developmental social isolation on immune function. On the other hand, when a chronic stressor such as social defeat was combined with social isolation, animals exhibited social avoidance when tested on post-natal day 41. However, such behavior was not observed in some of the non-isolated (group-housed) animals exposed to chronic social defeat, thus supporting the idea that social support is a resilience factor. The effectiveness of a pharmacological intervention (intranasal oxytocin) has also been examined in adult Titi monkeys exposed to temporary separation from pair mates (Arias del Razo et al.). The protective effect of chronic treatment of oxytocin (daily intranasal oxytocin dose from 12 to 18 months of age) was clearly demonstrated in that treated animals exhibited a lower level of distress when exposed to separation compared to control animals chronically treated with saline. However, although cortisol levels increased in response to separation, these hormone levels did not differ between the chronically treated and control animals.

In conclusion, despite the limitations associated with each of the studies and the diversity of findings, taken together, the findings point to the complexity of the relationship of loneliness and social isolation with neuroendocrine and immune function. This calls for further research with increased attention to potential moderators such as sex and age because males have been found to be more susceptible to the negative mental health consequences of loneliness [e.g., depressive symptoms (Liu et al., 2020); low satisfaction with life and low resilience (Zebhauser et al., 2014); risk for developing dementia (Zhou et al., 2018)] and the negative health effects of loneliness seem to increase with age (e.g., Hawkley and Cacioppo, 2007). In addition, factors blunting the negative effects of isolation or separation such as social support (Sailer et al.) and oxytocin (Arias del Razo et al.) should also be given due attention. Further studies focused on the complexity of neural, physiological, and behavioral interactions, and interdisciplinary research with human samples and animal models (Crespi et al.; Vitale and Smith) will advance our understanding of social isolation and loneliness. Hopefully, this knowledge will promote more sensitive public health policies fostering early intervention approaches to tackle the negative health consequences associated with loneliness and social isolation.

## Author contributions

JL contributed singly to summarizing the papers in this collection and writing of this editorial. All authors contributed to the article and approved the submitted version.

## Funding

CV was the principal investigator of a project funded by Spanish Ministry of Science, Innovation and University (RTI2018-094627-B-I00). AG was supported in part by the National Institutes of Health (HL147179). The research input from JL was supported by General Research Fund of the University Grants Committee of Hong Kong (number 9042922).

## Conflict of interest

The authors declare that the research was conducted in the absence of any commercial or financial relationships that could be construed as a potential conflict of interest.

## Publisher's note

All claims expressed in this article are solely those of the authors and do not necessarily represent those of their affiliated organizations, or those of the publisher, the editors and the reviewers. Any product that may be evaluated in this article, or claim that may be made by its manufacturer, is not guaranteed or endorsed by the publisher.
